# Balancing a Surgical vs. Non-surgical Approach in Perforated Appendicitis

**DOI:** 10.7759/cureus.71197

**Published:** 2024-10-10

**Authors:** Harriet Kaye Austin, Aron S Mcguirt

**Affiliations:** 1 Medicine, University of Central Florida College of Medicine, Orlando, USA; 2 General Surgery, Bay Pines Veterans Affairs (VA) Healthcare System, St. Petersburg, USA

**Keywords:** acute complicated appendicitis, appendiceal phlegmon, conventional appendectomy, nonoperative therapy followed by interval appendectomy, paralytic ileus

## Abstract

Appendicitis is the inflammation of the appendix, one of the common causes of acute abdomen that may indicate emergency abdominal surgical intervention. However, when complicated by perforation, phlegmon, and/or abscess formation, the standard and traditional treatment with the approach for a surgical intervention has been questioned regarding its risks and benefits. In this case presentation, our patient, who presented with perforated appendicitis complicated by phlegmon formation and ileus, was managed with a conservation approach through antibiotics and percutaneous drain placement. Although a conservative, non-surgical approach with interval appendectomy has become more of the popular approach in developed countries for complicated appendicitis due to increased morbidity in immediate appendectomy due to inflammation-associated adhesions, we wanted to present a case where a conservative approach can still lead to extended hospital stays due to complications.

## Introduction

Appendicitis is typically caused by the outflow obstruction of the appendiceal lumen, commonly from a fecalith. According to the literature, fecaliths were found to be associated with acute appendicitis in approximately 23.5% of patients and 24.9% of patients who had perforated appendicitis [1]. With the loss of outflow, the obstructed appendix can serve as a nidus for enteric bacterial overgrowth leading to inflammation and the appendiceal and subsequent inflammation of the parietal peritoneum [2]. If arterial supply becomes compromised, the appendix wall can become ischemic and lead to perforation two to three days after initial symptom onset. Perforation can then be complicated with abscess formation or peritonitis due to inflammation of the peritoneum from spillage.

The traditional standard approach for appendicitis has been appendectomy, which has evolved from open to laparoscopic/robotic, but recommended management continues to evolve as medical innovations continue to revolutionize the practice of medicine [3]. With the advancement in antibiotics, conservative management has become the popular approach for complicated appendicitis. Open appendectomies which are used as first-line treatment, are now rarely indicated as the initial approach. According to the literature, laparoscopic appendectomies have decreased the incidence of infection, length of hospital stay, earlier return to daily activities, and postoperative complications compared to open appendectomies [2]. More recently, a rapidly popular approach for appendicitis is the use of antibiotics, most especially for complicated appendicitis due to the increased risk for the morbidity of adjacent bowel injuries in immediate surgical intervention. Although conservative management is the current standard of care, there continues to be a debate in terms of patient outcomes in terms of quality of life, morbidity, and recurrence rates, along with cost-efficiency between surgical and non-surgical approaches.

## Case presentation

**Figure 1:**
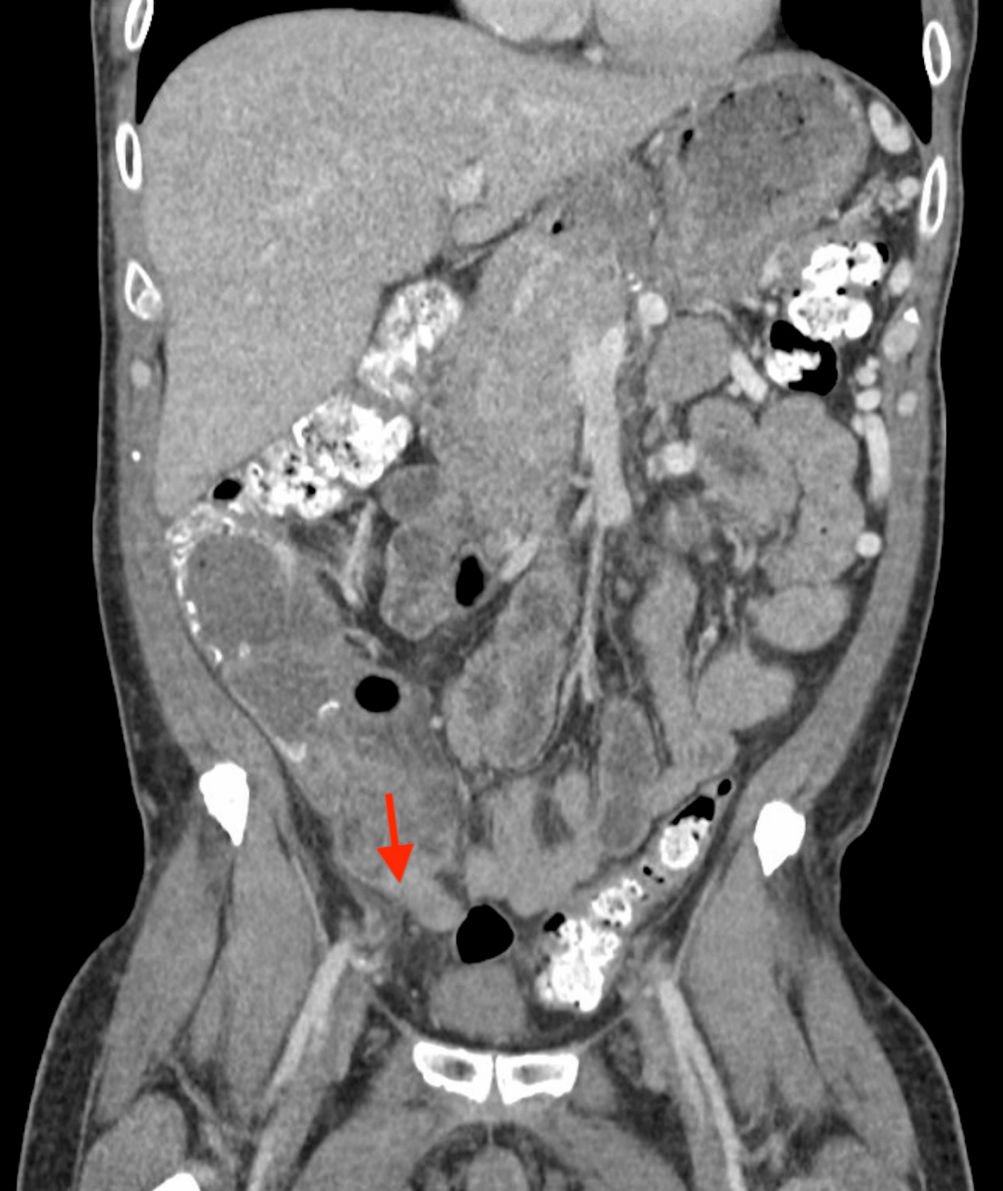
CT abdomen (coronal view) findings showing appendiceal thickening w/ surrounding inflammation in RLQ consistent with appendicitis CT: computed tomography, RLQ: right lower quadrant

**Figure 2:**
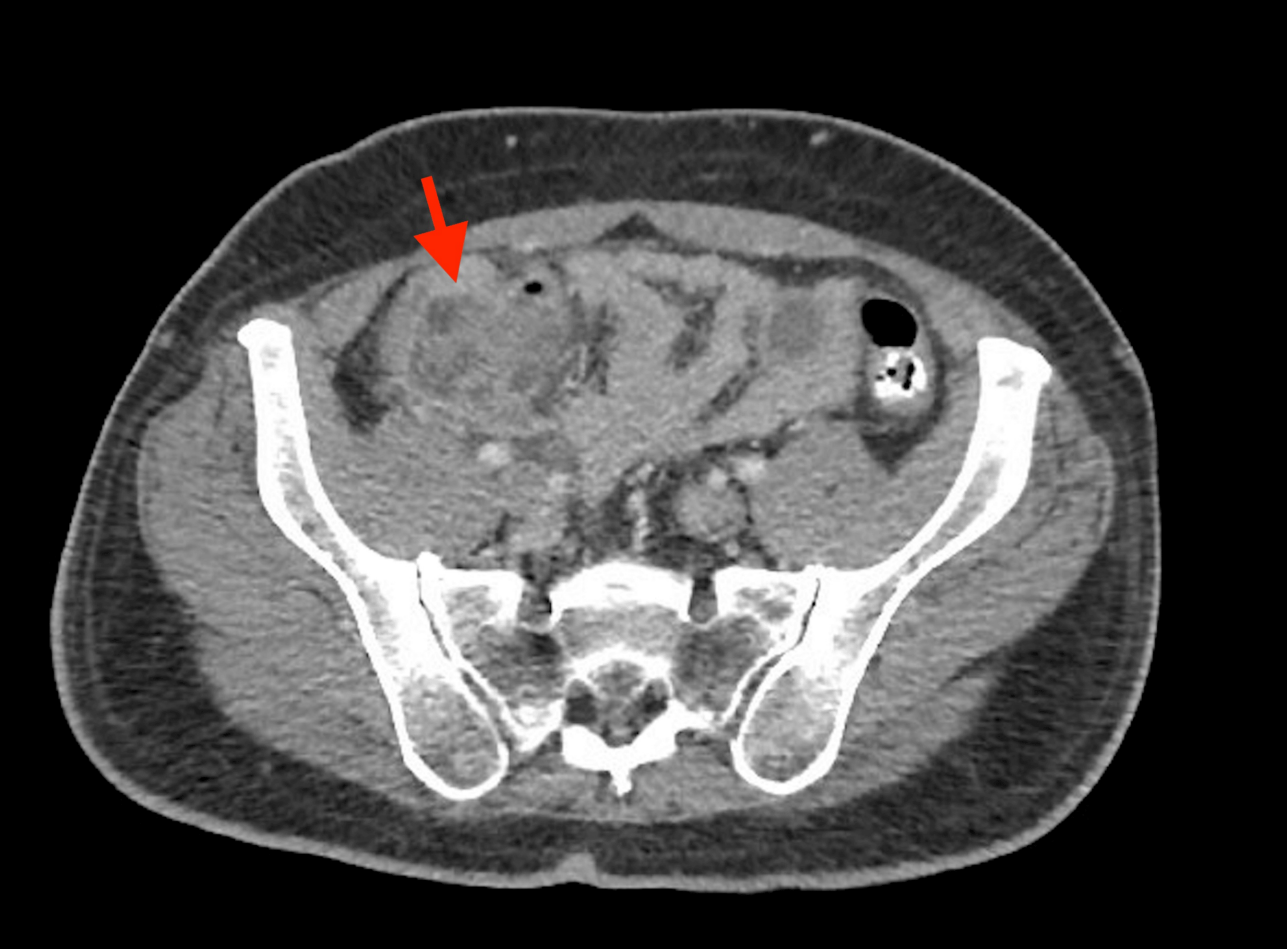
CT abdomen (axial view) findings showing appendiceal thickening w/ surrounding inflammation in RLQ consistent with appendicitis CT: computed tomography, RLQ: right lower quadrant

A 58-year-old African American male with a past medical history of type 2 diabetes mellitus, hypertension, and cerebrovascular accident in 2022 with no residual deficits presented to the emergency department due to a one-day history of the localized, dull right lower quadrant (RLQ) abdominal pain with three episodes of watery diarrhea, which has progressively worsened from a 4 out of 10 to a 7 out of 10 (Figures 1-2). He presented febrile with a temperature of 103, elevated blood pressure, and tachycardia. On exam, there was RLQ tenderness on palpation without distention and signs of peritonitis. He presented with leukocytosis of 17 x 10^9^/L and hyperlactatemia of 4.4 mmol/L with perforated appendicitis with moderated volume fluid in RLQ and diffuse bladder wall thickening on CT abdomen (Table 1). He was subsequently admitted to the surgical intensive care unit due to sepsis presentation and placed on NPO with multi-modal pain management, IV fluid resuscitation, and IV Zosyn and vancomycin.

**Table 1:** Lab values on admission + National Board of Medical Examiners (NBME) laboratory values [4], * American Board of Internal Medicine (ABIM) laboratory test reference ranges [5], WNL: within normal limits, WBC: white blood cell

	Normal reference range	Day 1	Day 5	Day 6	Day 10
WBC^+^	4.5-11.0 x 10^9^/L	17 x 10^9^/L	16.2 x 10^9^/L	14.4 x 10^9^/L	WNL
Glucose^+^	<140 mg/dL	159 mg/dL	–	–	–
Lactate^*^	0.7-2.1 mmol/L	4.4 mmol/L	–	–	–

The following day, the patient underwent CT-guided aspiration of the RLQ abdominal wall with pertinent findings of dilated appendix and phlegmon formation. At the time, IR decided not to place a drain due to the small amount of fluid that was aspiration. The patient was then added to fluconazole while awaiting a culture of aspirated fluid collection. On the third day of admission, he reported having a one-time episode of emesis consisting of bilious contents of large volume. At the time, he did not show any peritoneal signs, with the last bowel movement occurring prior to admission. A nasogastric tube (NGT) was placed with a bilious output of 550 ml. Due to lack of improvement and per recommendations, the patient was advanced from Zosyn to meropenem along with increased fluconazole due to a leak from perforation.

On the fourth day of admission, the patient underwent CT-guided Jackson-Pratt (JP) surgical drain placement with an output of 15 cc of purulent fluid and pertinent findings of ileus and small bowel dilation of CT abdomen and pelvis. Fluid cultures showed growth of *Escherichia coli* and *Klebsiella* species. Over the next two days, NGT continued to drain 700-900 cc of bilious output with an improvement of leukocytosis from 17 on admission to 14.4 on day six of admission. However, abdominal X-rays showed findings of significant ileus vs. partial obstruction secondary to phlegmon. RLQ drain remained to have minimal dark serous output. Vancomycin was discontinued and started on ceftriaxone. By day eight of admission, the patient had his first bowel movement but remained significantly distended and mildly tender to palpation. The final culture report showed the growth of *Escherichia coli*, *Klebsiella pneumoniae*, *Clostridium innocuum*, and beta-lactamase-positive anaerobic gram-negative rods resembling *Parabacteroides distasonis*. By day eleven of admission, the patient was passing flatus, ambulating, and voiding and reported significant improvement in pain with mild abdominal distention. The NGT was removed as it was no longer indicated, and leukocytosis had resolved. Per additional recommendations from infectious disease, ceftriaxone was discontinued, and the patient was switched from IV meropenem to ertapenem. On day 13 of admission, the JP drain was removed due to minimal output, and the patient was discharged to home with the completion of an antibiotic course and outpatient follow-up.

## Discussion

Of the patients who present with acute appendicitis, 13-20% present with perforation, and management of patients with perforated appendicitis depends on the condition and nature of the perforation [3]. For stable patients, immediate appendectomy or nonoperative management were both reported to be safe approaches. Based on current literature, there was “very low-certainty evidence” suggesting early appendectomy reduces the abdominal abscess rate compared to delayed appendectomy for pediatric and adult patients with appendiceal phlegmon [6]. In the pediatric population, the use of percutaneous drainage with antibiotics and interval appendectomy in comparison to immediate appendectomy did not show clinically significant differences in costs, length of hospitalization, or recurrence rate [2]. However, for patients with long-duration symptoms and formation of phlegmon or abscess formation, immediate surgery has been associated with increased morbidity and difficult intraoperative management due to extensive dissection of dense adhesions secondary to prolonged inflammation. Although the literature does not recommend routine interval appendectomy for healthy young adults as they are less likely to have severe sequelae from appendicitis recurrence, shortcomings in current diagnostic strategies combined with delayed timely surgical care, especially for the 15-49% of patients initially managed nonoperatively who experience recurrent appendicitis, may have a negative psychosocial impact on the patient and/or their family, subsequently reducing quality of life [2]. On the other hand, adult patients over 40 years old are at increased risk for the presence of cecal or appendiceal neoplasm, prompting colonoscopy screening and interval appendectomy for specimen pathology [3].

Although there is a high success rate of nonoperative management of complicated appendicitis treatment of percutaneous drainage and antibiotics, studies show that there were no significant differences in hospital length of stay for patients who received nonoperative management compared to immediate operative management. However, the success of percutaneous drainage and antibiotics has allowed for a minimally invasive treatment approach that does not place the patient at risk for surgical complications that accompany appendectomy [7]. From the same study, patients who failed nonoperative management had extended hospital length of stay and were at high risk for open surgery with possible bowel resection and postoperative complications. Based on a study, factors that were associated with failure of nonoperative management were smoking, tachycardia, generalized abdominal tenderness, and abscesses that were less than 50 mm [8]. Another study found an inverse relationship between the likelihood of failure and symptom duration; patients with longer symptom duration had a reduced likelihood of failure of nonoperative management [9].

Management for complicated appendicitis continues to remain controversial, with current literature not showing strong evidence that supports or refutes the need for interval appendectomy in patients who are initially managed with a nonoperative approach [10]. Even with technological advancements and improved techniques in laparoscopic appendectomy, all types of surgeries come with risks for complications. Decisions for subsequent steps after nonoperative management, including interval appendectomy, should be made through shared decision-making between the patient and clinician based on the patient’s risk factors and state of well-being.

## Conclusions

There are risks and benefits associated with both nonoperative and operative management of complicated appendicitis. There is no clear algorithm with strong evidence favoring one approach more than the other. Treatment of stable patients presenting with complicated appendicitis requires consideration of comorbid conditions and risk factors for complications. More importantly, informing the patient of risks, benefits, and alternatives of treatment options to promote shared decision-making in opting for operative management or trial of nonoperative management should be prioritized to uphold patient-centered care. In this patient, his complication of ileus led to increased hospital stay; however, it is difficult to affirmatively state whether immediate operative management, which carries its own risks for complications, would have led to a better outcome. Despite the benefits of successful nonoperative management, not all patients may not capitalize from it. There is a need for better identification of patients who are more likely to fail nonoperative management to optimize treatment strategies and subsequently improve patient outcomes.
